# Medical Ideals Unrealised

**Published:** 1894-03-03

**Authors:** 


### The Hospital

An Institutional Journal of

The Medical Sciences and Hospital Administration, with

Vol. XV.—No. 388.] AN EXTRA NURSING SUPPLEMENT. [March 3, 1894.

### Medical Ideals Unrealised.

It cannot but be the experience of thousands of medical men—of all, in fact, who possess clear intelligence and profound human sympathies—that they realise in their own minds and inwardly protest against the wide extent of their own impotence. A man who had little acquaintance with the inner problems of medicine, but a deep knowledge of the almost insuperable difficulties which lie in the pathways of all kinds of human advancement, formulated this dictum—"To be weak is the true misery." It was Carlyle who discovered that inner cause of discontent. This is what the practising doctor, whose mental susceptibilities are not blunted by routine to absolute and incurable dulness, must often feel when he is brought face to face with some of the more serious obligations of our art.

Of such diseases as consumption and cancer we need not speak in this connection; it is recognised by all honest practitioners that methods of cure for those diseases, upon which the practitioner can place assured reliance, are not known. Efforts may be and are constantly made in individual cases, and a certain amount of success is the result. But of assured methods in which the profession as a whole can trust we know none. It is not to our impotence in these directions that attention is now asked. There are diseases which are in their own nature curable, which, in fact, in many instances tend to undergo spontaneous cure, which, nevertheless, in many other instances are not cured at all, and which, in the fact of their non-cure, reflect the smallest possible amount of credit upon the physician's art. As illustrations of these may be mentioned the whole class of generalised inflammations of internal organs. A modern practitioner is called to a case of acute double pneumonia. What does he do for his patient which unaided and unhindered nature would not do? It is true he makes a correct diagnosis; he realises with the utmost clearness in his own mind the actual facts of the case; he sees with painful distinctness the swollen arterioles, the engorged capillaries, the compressed bronchioles and alveoli; the choked condition of the air passages, the paralysed nerves, the proliferating epithelium, the distended lymphatics, the whole picture, in fact, of an organ suddenly suspending its activity and functions in powerless prostration before the onslaught of a contagious poison or a paralysing chill. Or is it a case of acute nephritis to which the practitioner is called? Here, if possible, the demand for immediate relief is still more urgent, and the task of carrying the patient through his illness to the period of ultimate and complete restoration of kidney integrity and service still more difficult. The unparalleled complexity of structure in the kidney, the careful packing of blood vessels, and of secreting and excreting tubules for the economisation of space, the hourly importance to the whole organism of the functions performed by the kidney—all these, associated with the acutely congested condition, present to the mind of the physician-pathologist a task so urgent, so delicate, so difficult, so almost impossible, that if it were not familiar it would be appalling.

In the face of problems like these, who can wonder that as knowledge in pathology has become clearer and more profound, mere expectancy in treatment should have become more universal and more approved? The old-fashioned practitioner who always "did something" was bold in proportion to his ignorance. The physician of to-day, who adds a profound knowledge of pathology to his other accomplishments, is too often timid in the exact ratio of his knowledge. But "expectancy," we submit, is not what the patient and his friends "expect" of us, and it is certainly a poor ideal to set before the ardent student of pathology in our progressive epoch. If this be all which we have gained, or are to gain by the progress of physiological and pathological science, we can sympathise with those who aver that the old times when medicine was a mere clinical art were better for the patient than these in which it attempts to be an exact science.

In such cases as we have considered, of acute inflammations of internal organs and of organs whose integrity is essential to life in its fullness, we ought to be able to do something of a promptly effective kind towards the two objects of immediately relieving the sufferings of the patient, and of conserving for future and lifelong service the structural integrity of the inflamed organ. If we cannot do anything of an effective kind in the direction of these two objects, what is our treatment but the feeblest expectancy, however we may disguise it by more hopeful names? Something we should do—but what? It must be rational; it must be physiological; it must have the promise and potency of real help in it—of help in the form of present relief, and of help in the form of the preservation of the structural and functional integrity of the affected organ. We look round among all the modern resources of the physician's art for some drug which shall render us' these services in the case of an acute inflammation of the kidney or of other internal organ. What of approved and assured value do we find among the remedies of recent times? Nothing, absolutely nothing at all! The old fashioned practitioner would have made excellent use of calomel: would he have done right? Undoubtedly he would! Or he would have had still more confidence in a mode of treatment now universally neglected: he would have tried blood letting! Would he have done right? An answer to this question is being anxiously sought by some of the most truly scientific and deep-seeing clinicians of our questioning period.

Would the withdrawal of a considerable quantity of blood give immediate relief to the suffocative breathing of a patient in the very early stages of simple acute pneumonia? Most certainly it would! Would one, or two, or more withdrawals of blood so lower the dangerous pressure in the vessels and lymphatics of the affected organ that the products of inflammatory action would be greatly diminished, and structural integrity be correspondingly conserved? There are excellent physiological reasons for believing that these results would ensue. If in the lung there would be this immediate relief, with the almost certain conservation of structural integrity, still more might we expect, and still more do we need to strive for, such results in the kidney, where the organ itself is so much smaller and denser, and the [difficulty of finally getting rid of the products of inflammatiory action is so much more markedly pronounced.

Not science for the sake of science must be the physician's ideal, but science for the sake of relief; and, above all, science for the sake of ideally perfect cures. A chronically congested kidney, a long-standing chest catarrh, an unyielding and uncured gastritis—these constitute a standing reproach to the physician which he cannot but take to heart. The architect rears his church or his theatre: builds it and puts on the stone of final completion. It is a finished work. Similarly the engineer plans and constructs his bridge or his tunnel, and the day arrives when the last stroke of the workman's hammer is heard. If physic is to retain and improve its position as a scientifically-based art of the highest service to mankind, it too must aim at complete ideals—ideals of definite and adequate relief, ideals of structural and functional restoration which shall not fall short of finished perfection.

